# Zebrafish as a Model System to Study the Mechanism of Cutaneous Wound Healing and Drug Discovery: Advantages and Challenges

**DOI:** 10.3390/ph14101058

**Published:** 2021-10-18

**Authors:** Ruth Naomi, Hasnah Bahari, Muhammad Dain Yazid, Hashim Embong, Fezah Othman

**Affiliations:** 1 Department of Human Anatomy, Faculty of Medicine and Health Sciences, Universiti Putra Malaysia, Serdang 43400, Malaysia; ruthmanuel2104@gmail.com (R.N.); haba@upm.edu.my (H.B.); 2 Centre for Tissue Engineering and Regenerative Medicine, Faculty of Medicine, Universiti Kebangsaan Malaysia, Cheras, Kuala Lumpur 56000, Malaysia; dain@ukm.edu.my; 3 Department of Emergency Medicine, Faculty of Medicine, Universiti Kebangsaan Malaysia, Cheras, Kuala Lumpur 56000, Malaysia; hashimembong77@ukm.edu.my; 4 Department of Biomedical Sciences, Faculty of Medicine and Health Sciences, Universiti Putra Malaysia, Serdang 43400, Malaysia

**Keywords:** zebrafish, wound healing, in vivo, signaling mechanism, advantages, challenges, drug discovery

## Abstract

In humans, cutaneous wounds may heal without scars during embryogenesis. However, in the adult phase, the similar wound may undergo a few events such as homeostasis, blood clotting, inflammation, vascularization, and the formation of granulation tissue, which may leave a scar at the injury site. In consideration of this, research evolves daily to improve the healing mechanism in which the wound may heal without scarring. In regard to this, zebrafish (*Danio rerio*) serves as an ideal model to study the underlying signaling mechanism of wound healing. This is an important factor in determining a relevant drug formulation for wound healing. This review scrutinizes the biology of zebrafish and how this favors the cutaneous wound healing relevant to the in vivo evidence. This review aimed to provide the current insights on drug discovery for cutaneous wound healing based on the zebrafish model. The advantages and challenges in utilizing the zebrafish model for cutaneous wound healing are discussed in this review. This review is expected to provide an idea to formulate an appropriate drug for cutaneous wound healing relevant to the underlying signaling mechanism. Therefore, this narrative review recapitulates current evidence from in vivo studies on the cutaneous wound healing mechanism, which favours the discovery of new drugs. This article concludes with the need for zebrafish as an investigation model for biomedical research in the future to ensure that drug repositions are well suited for human skin.

## 1. Introduction

Any injury to the skin is termed as a cutaneous wound. It is predicted that approximately 2% of the population will experience a chronic wound in their lifetime. Epidemiologically, in the United States there are 6.5 million subjects with chronic wounds that exist up to today [1], while in Malaysia more than 13,000 subjects have visited wound care units since 2003 [2]. Considering this awful situation, researchers are always attentive to study the underlying mechanism of wound healing to prevent further complication and to ease the healing mechanism. For the past few decades, the use of zebrafish (*Danio rerio*) as a model system to study various pathologies has recently caught the attention of researchers worldwide [3]. Zebrafish are tropical freshwater fish that live in the rivers of South Asia’s Himalayan zone. They are categorized as a teleost and belong to the Cyprinidae family and the Actinopterygii class [4]. Numerous major features of the zebrafish in comparison with mammals further attract researchers to explore the root of pathology using zebrafish. Zebrafish embryos grow externally, rapidly, and in a visible manner [5]. This allows the scientist to track the life cycle of zebrafish easily. The major organs of baby zebrafish start functioning as early as 5 days, and they are able to reproduce all year where females have the capability to generate hundreds of embryos per week [6].

The ability to regenerate itself, particularly the organs, make zebrafish a high-potential research organism. Nonetheless, the small body feature, the ability to produce a large number of offspring in a short duration, the extremely short period for growth and reproductive cycle, the transparent embryo, and the similarity of genes and major organs with humans further add to the list of benefits for scientists to use this tiny organism as a research model [7]. Researchers have defined zebrafish as a canonical vertebrate due to their shared aspects with humans. Resultantly, many laboratories exploit the biological system of the zebrafish to investigate the undefined pathologies and drug discovery [8,9,10,11,12,13,14]. This includes the development of disease-related assays and gene homologs related to human diseases [15]. In this context, zebrafish are an emerging organism that is being studied as a tool for cutaneous wound healing and also in the field of drug discovery. In this review, we scrutinized the recent data that support the development of flavonoid compounds as a new drug for cutaneous wound healing. The review also recapitulates the skin structure of zebrafish, the advantages and challenges of using zebrafish as a cutaneous model, and toxicity studies concentrating on drug discovery (flavonoid compounds) for wound healing.

## 2. Biology of Zebrafish

A zebrafish usually appears with an elongated body measuring up to 6 cm in length. In the adult phase, the head is usually slightly compressed at the dorsal end with a mouth that forms upwards. It does not have any teeth present orally, but its teeth are commonly linked in the 5th brachial arch, which is comprised of a layer of dentine, a pulp core, and an enamel coating. Barbels are two pairs of epidermal sensory appendages found in zebrafish. The rostral, which are also known as nasal barbels, grow to the orbit’s anterior boundary with another lengthy maxillary barbels on either side of the mouth. The presence of white silver lines on each side of their body and the pale yellow in the dorsal and belly region make the zebrafish to appear as if golden in colour [16]. A female zebrafish is able to generate hundreds of eggs in a single spawning. The eggs, normally with diameters of around 0.7 mm, are dropped onto a substrate. The developmental stage begins when the eggs are fertilized by the male sperm. In case of the absence of male sperm, the water itself can activate the eggs. However, the developmental stage for unfertilized eggs does not proceed beyond the cleavage stages [17]. If the eggs have been fertilized within 72 h of spawning, zygote formation will eventually begin followed by division of eggs in the cleavage stage, resulting in 16 to 64 cells. During this stage, a hollow ball-shaped embryo consisting of many layers of cells, which is also known as a blastula, will be produced. This will thereby be surrounded by a cavity defined as the blastocele. This stage may last from 40 min to 2 h [18].

**Figure 1:**
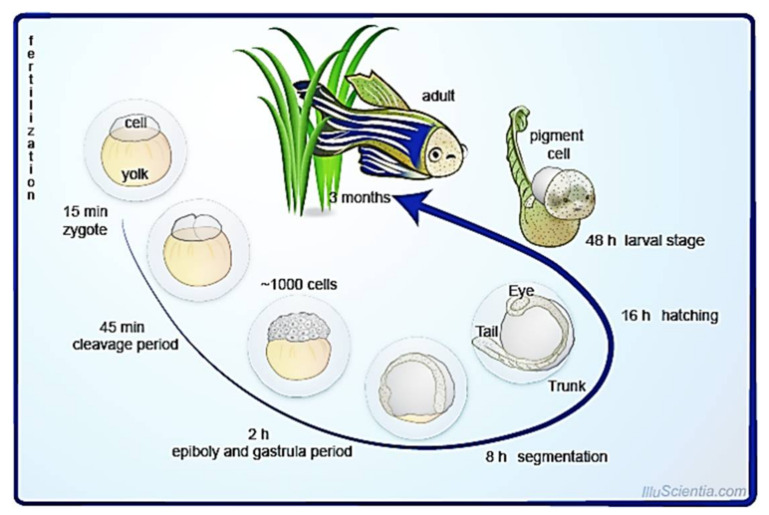
Developmental stages of zebrafish. Figure is reused under the permission obtained from Creative Commons Attribution-ShareAlike License [20].

The developmental stage will then progress into the gastrulation stage. In this stage, three germ layers will be formed. Herein, it prepares for the whole organism formation. The developmental process continues with segmentation and the pharyngula phase before hatching. During this phase, significant growth of organs such as the head and the tail occurs. This phase prepares the offspring for hatching. Depending on the thickness of the chorion, hatching may last between 48 to 72 h. The larvae are approximately 3-mm long once they are hatched. The larvae cling to hard surfaces via secretory cells on the surface of their heads. Within the next 3 days, other anatomical parts will begin to form via the process of morphogenesis. Post 7 days of fertilization, the organism would be able to swim, whereas the organism is said to be fully matured and able to reproduce by week 10. All organisms will develop ovaries before being differentiated into male gonads [19]. Figure 1 summarizes the developmental stages of zebrafish.

### 2.1. Skin Structure of Zebrafish

The skin development of zebrafish is based on transverse portions of the caudal peduncle, at the level of the anal fin area [21]. Fully developed skin structure in zebrafish is visible on the 6th day post-fertilization, and the epidermis and dermis are formed as early as the 1st day post-fertilization. The surface of the zebrafish consists of well-demarcated keratinocytes and developing microridges with the appearance of a spicule-like extensions that is observable on the first day post-fertilization. A basement membrane comprised of hemidesmosomes, which divides the connective tissue stroma and two cell layers in the epidermis, is evident during the 6th day post-fertilization. Meanwhile, the presence of numerous fibroblastic cells, collagenous stroma, and well-developed rough endoplasmic reticulum were obvious at the dermal region [22]. In sum, within only 6 days post-fertilization, the well-defined layers of the epidermis, the dermis, and the basement membrane separating multilayer of the epidermis from the underlying collagenous stroma are fully formed in zebrafish.

On the 10th day post-fertilization, the skin of the zebrafish is only a short distance away from the well-differentiated muscle cell surface. The epidermis’s basal-layer cells will be densely packed with microfilament bundles. A layer of dermal endothelial cells commonly lines the deep surface of the primary dermal stroma, most of which display significant protein synthesis activity. Upon reaching the 26th day, the skin usually comprises four cell layers with improved thickness (approximately 14 µm). In such a condition, a clear cytoplasm with the absence of organelles is visible in the superficial layer of the cells, while the presence of goblet cells is observable between the intermediate layers of the cells. By the 30th day post-fertilization, at particular loci, dermal papillae will be formed. During this period, fully developed zebrafish owning its own unique scales near to the epidermal cells due to sonic hedgehog expression can be witnessed. The scales usually will be developed inside the papillae [21]. Besides, zebrafish skin features a neural crest-derived pigment cell system comprised of melanocytes, which eases the application of this organism as a model for cutaneous wound healing [23].

### 2.2. Physiology of Cutaneous Wound Healing in Mammals

Wound healing is a complex process involving a few phases. In mammals, the healing at the injury site is achieved with four overlapping phases. This starts with the hemostatic phase in which the static of blood takes place, followed by the inflammatory phase, which is preceded with the proliferative or granulation phase and which ends with the remodeling or maturation phase. In the hemostatic phase, the platelet will come into contact with the collagen in the endothelium of the blood vessel, resulting in the activation of platelets, causing the platelets to aggregate at the injury site. Simultaneously, an enzyme, thrombin, will stimulate the fibrin mesh formation. The meshwork of fibrin at this site ensures a stable clot by strengthening the platelet clump together. Once there is a stable clot, neutrophils will shield the wound by detecting and removing any foreign bodies. This is a crucial step in ensuring the formation of the wound bed [24]. The process is followed by macrophage-removing debris via a process called phagocytes. Usually, the macrophage and neutrophils hit the peak by 24 to 48 h upon injury and are drastically reduced over the following three days. Once the wound site is safe and clean, secretion of growth factors and proteins will be stimulated in order to attract further immune cells to expedite the tissue repair [24].

**Figure 2:**
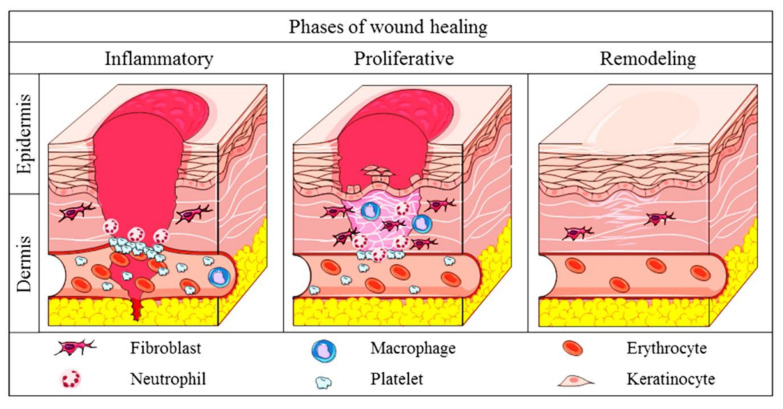
Stages of cutaneous wound healing in mammals. Figure is reused under the permission obtained from Creative Commons Attribution-ShareAlike License [27].

The healing phase will then be continued with the proliferative or granulation stage, where the injured site will be epithelized or covered with new skin. This stage can be further broken down into three categories, namely, filling of the wound, marginal contraction, and re-epithelialization. During this phase, granulation tissue will populate the wound bed with connective tissue. When this happens, new blood vessels will start to form, and the marginal side of the wound will begin to contract and will be dragged toward the center of the injury site. Resultantly, the epithelial cells will emerge from the wound bed or margin [25]. Now, in a leapfrog pattern, these cells will start to migrate across the wound bed, leading to the coverage of the wound site with the epithelium. This phase usually materializes from 4 to 24 days depending on the severity of the wound. The healing mechanism will then be preceded with the maturation phase, where the newly formed tissue will gradually gain durability and strength. The collagen fibers will start to re-organize, allowing the tissue to remodel and mature [26]. Figure 2 shows the mechanism of cutaneous wound healing.

### 2.3. Comparison of Wound Healing: Mammals and Zebrafish

In mammals, wound-healing stages may involve several continuous and overlapping processes starting with blood clotting, inflammation, re-epithelialization, granulation tissue formation, and, finally, remodeling [27]. Zebrafish share the same features in the wound-healing phase except for the absence of the blood-clotting phase. However, the duration of each phase slightly differs in comparison to those of the mammals. Unlike mammals, zebrafish do not really have neutrophils and macrophages. In substitutes, myeloperoxidase-expressing cells are known to be neutrophils, while lysozyme-expressing cells are macrophages due to the lineage specificity of the lysozyme promoter [28]. Thus, macrophages tend to stay in the wound during the healing stage longer compared to neutrophils. Neutrophils possess the ability to survive the early inflammatory phase and to later migrate back into the peripheral bloodstream. Unlike mammals, in zebrafish, re-epithelialization is the first phase [29] and begins immediately, even before inflammation, due to the absence of granulation tissue or blood-clotting inflammation. As a result, it appears that re-epithelialization is mainly independent of extracellular-matrix proteins provided by the blood clot or granulation tissue. This might be owed to the re-epithelializing epidermis’s main influence of tissue-autonomous extension movement [30], and usually this phase lasts less than 10 h [29].

**Figure 3:**
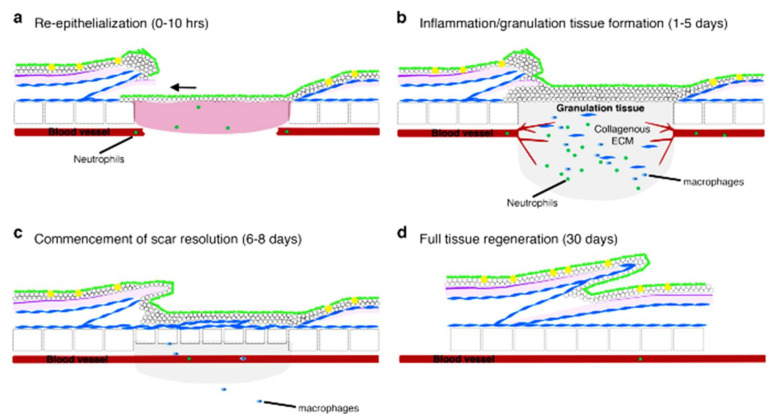
Mechanism of wound healing in zebrafish. Figure is re-used under the permission obtained from licensed under a Creative Commons Attribution 4.0 International License [29].

These will then be proceeded with neovascularization and the deposition of collagen below the re-epithelialized site. The granulation tissue and inflammatory cells are drastically decreased by day six after wounding, and dermal thickenings will begin to replace lost scales; the tissues are said to be entirely repaired by day 30 following wounding [31]. Interestingly, recent data prove that the inflammatory response has little or no effect in the re-epithelialization process of mammals [29]. Conversely, unlike mammals, neutrophils and chemotactic signals from macrophages have little effect on the re-epithelialization of wound debridement in zebrafish [32]. The rapid healing mechanism of zebrafish could possibly be due to the presence of live cells that are capable of cell elongation and radial intercalation coordinated by Rho/Rock and the TGF signaling pathway [33]. Figure 3 shows the phases of wound healing in zebrafish.

## 3. Zebrafish as a Model to Study the Mechanism of Cutaneous Wound Healing

The basic principles of the wound-healing mechanism are conserved between humans and zebrafish due to the similarity of their skin structure. In relation to this claim, in both the embryo and larval phases, the zebrafish’s skin is already comprised of periderm at the superficial layer, an epidermis bilayer in the middle, and a basal layer attached to the basement membrane [34]. Its multilayer of the epidermis will be formed following the 25th day post-fertilization during a process called metamorphosis. Simultaneously, fibroblasts penetrate the dermis, taking over collagen production from basal keratinocytes and forming localized thickenings known as dermal papilla to start scale creation [35]. This proves the similarity between human skin architecture, which enables the possibility of enhancing this species as a model for cutaneous wound healing. Due to this, over the past decade, zebrafish have already been established as a model for cutaneous wound healing. Here, we briefly introduce the signaling mechanism and the underlying mechanism of healing discovered during utilization of zebrafish as a model for the cutaneous wound-healing model. Table 1 summarizes the data obtained during in vivo investigation of zebrafish as a wound-healing model.

Lisse et al. (2016) [36] and Seo et al. (2017) [37] modelled zebrafish to study epidermal wound healing. The outcome obtained in both studies correlate with one another, although the used compound varied. Both obtained results showed complete filling of the cavity, re-surfaced epidermal cells, and well-formed skin; immune cells were seen near the wound as early as day four. The outcome obtained proves that the activation of the EGF, FOXO1, and IKKα pathways are essential, underlying the signaling mechanism in cutaneous wound healing. This is further supported by Caraguel et al. 2016 [38] who witness similar results in their in vivo investigation. Upon identifying the migration of cells towards dermal region due to increased level of EGF production in the basal epidermal, they claimed that the result acquired by Lisse et al. (2016) [36] is scientifically provable.

Conversely, Richardson et al. (2013) [34], Caraguel et al. (2016) [38], Xiong et al. (2018) [39], Vimalraj et al. (2018) [40], Noishiki et al., (2019) [41], Liu et al., 2020 [42], and Edirisinghe et al. 2020 [43] studied the mechanism of full-thickness wound healing using zebrafish. Despite differences in the strain used, the results from 100% of the studies showed promising results. This is because all the study outcomes showed rapid rate of re-epithelialization, formation of the neo-epidermis, fully re-stratified wound epidermis, dermal compartments, and completely recovered subcutaneous adipocytes; re-formation of scales were seen as early as day two. A study done by Vimalraj et al. (2018) [40] exhibited mitotic activity, resulting in cell division in the skin layer, a similar mechanism noted in human wound healing. This indicates the comparable genome structure of zebrafish and humans, where zebrafish shares 70% of the same genes with humans. Nonetheless, similar to the mammalian wound-healing mechanism, the zebrafish model exhibits the formation of granulation tissues and inflammatory responses including the invasion of fibroblasts, IL-1β, TNF-α, SOD, and catalase [34].

### Signaling Mechanism in Zebrafish Model for Wound Healing

The complexity of zebrafish develops from one cell to a complete multicellular organism during metazoan embryonic development, resulting in the modification of the mode of communication between these parts. The interactions are said to be very limited during the early embryo formation, which exists only along two axes. However, during the primordia formation, the differentiation in the body parts stimulates numerous interactions, which involve multiple signaling pathway activations [44]. These developmental stages can be used to analyze underlying the molecular pathway in wound healing. In the wound healing phase, the activation of fibroblast-growth-factor (FGF) signaling in mammalian species is essential for the formation of granulation tissue and re-epithelialization. Stimulation of angiogenesis is observed in in vivo assays of adult zebrafish due to the expression of FGF triggering the direct effect on fibroblasts, which proves that the underlying genetic and mechanistic principle in cutaneous wound healing is conserved in zebrafish alike in the mammalian species [34]. A slight modification in the method of in vivo assay of zebrafish reveals the evidence of the H_2_O_2_ downstream signal transduction pathway activation including EGF, FOXO1, and IKKα [36]. This stimulates the migration of immune cells towards keratinocytes and sensory axon repair, while promoting a cytoprotective effect in the wounded region, which thereby prevents oxidation damage in the exposed region [45].

In contrast, stimulation of the epidermal-growth-factor (EGF) pathway was observed by Caraguel et al. (2016) [38] in the zebrafish in vivo assay. In mammals, a peak in EGF is noticeable 15 min upon injury, and usually this is localized at the region of migrating epithelial cells only. In this condition, EGF will, in turn, regulate the transforming growth factor beta (TGFβ) through ERK1/2 and EGFR signaling [46]. As a result, injured cells will bind to the EGF receptor, causing dimerization of the ligand-induced receptor. This will, in turn, trigger the activity of intrinsic proteins such as tyrosine kinase. Thus, the downstream signaling pathway will be stimulated, causing dramatic changes in the intracellular calcium levels, a rise in the glycolysis process, and stimulation of the gene transcription. This leads to DNA synthesis and cell proliferation at the wound site, thereby accelerating the healing process [47]. Similarly, a study by Richardson et al. (2016) [48] indicates that regulation of the TGFβ/integrin and the Rock/JNK pathways may accelerate the re-epithelialization process.

Besides, Edirisinghe et al. (2020) [43] and Vimalraj et al. (2018) [40] investigated the molecular pathway involved in wound healing using adult zebrafish. Interestingly, both of them noticed the upregulation of the Wnt/β-catenin, which resulted in rapid remodeling of the epidermal tissue. Upregulation of the Wnt/β-catenin is essential during the proliferative phase of wound healing. This is because stimulation of the Wnt/β-catenin molecular pathway will stimulate the β-catenin to accumulate within the nucleus, causing genes responsible for cell proliferation to transcribe. As a result, there is a noticeable increase in dermal cell proliferation, motility, invasiveness, and wound size reduction [49]. This signifies that 100% of the in vivo research outcome indicates that zebrafish can be modeled to study the molecular mechanism of cutaneous wound healing.

## 4. Zebrafish as a Model for Drug Discovery in Cutaneous Wound Healing

Zebrafish have been identified as a viable in vivo model for drug discovery related to cutaneous wound healing, regeneration, and angiogenesis [50]. There are many reasons why zebrafish have become a convenient alternative for drug discovery, some of which include the fact that the model allows for rapid, non-invasive, and direct observation of results following a chemical intervention [34]. In addition, researchers may just need to introduce treatment interventions into the water to alleviate the healing process in cutaneous injury. This actually prevents further injury to the organism as there is no need to introduce drugs intravenously or injections to study the interference of drugs in healing the wounds [51]. In addition to the application of the zebrafish model to study the efficacy of potential drug targets in wound healing, it is also used for testing the toxicity of available drugs used in treating wounds. In these, various chemicals, metals, and natural compounds are being introduced to the zebrafish to investigate the efficiency in accelerating the wound-healing mechanism. Several promising results seen through their in vivo trials (Table 2) are surely an initiator to develop wound-healing drugs.

### 4.1. Natural Compounds 

Several natural-compound extracts have modelled zebrafish for wound healing. These natural products contain medicinal properties that can facilitate the wound-healing process. In these, *Panax ginseng* extract contains bioactive compounds such as ginsenoside Rg1, which may exhibit anti-inflammatory and anti-oxidant properties at the wounded region [52,53]. Such properties have proven to enhance healing by increasing the rate of collagen synthesis at the injury site [54]. In the study by He et al. (2020), they investigated the anti-inflammatory effects of Rg1 using a tail fin amputation of the Zebrafish larva [53]. In the study, they identified that the anti-inflammatory property of Rg1 is comparable to beclomethasone, in which both substances attenuate neutrophilic inflammation at the amputation site. The attenuation of neutrophil recruitment to the wound will suppress the radical oxygen species (ROS) production that will eventually enhance wound-healing activities [55]. Moreover, work by He et al. (2020) revealed that Rg1 does not inhibit tissue regeneration, which will give their benefit over beclomethasone for a more effective wound-healing process [53]. Other than that, a study by Sung et al. (2017) showed that Rg1 also promotes angiogenesis, which is one of the important components in the wound-healing process [56]. The study demonstrated a significant increase in sub-intestinal vascular growth of the zebrafish embryos after being incubated with *Panax ginseng* extract (500 μg/mL).

The caudal fin amputation technique has also been established in a study investigating the role of *Curcuma longa* extract (CLE) as wound-healing agents [57]. The study demonstrates that the topical application of CLE accelerates the regeneration process of the amputated fin in adult zebrafish. This healing efficacy of CLE is contributed to by several biological activities including anti-inflammatory, anti-microbial, anti-oxidant, and anti-apoptotic activities [58,59,60]. In the study by Kim et al. (2021), they suggested that the waterborne exposure of CLE to zebrafish embryos provides a strong protective effect on H_2_O_2_-induced oxidative stress. These antioxidant effects of CLE are suitable as an alternative therapy in oxidative-stress-induced cellular damage such as diabetic wound healing.

Propolis is another natural compound that appears as an alternative remedy for wound care. Propolis is known to have anti-oxidant and anti-inflammatory activities that could neutralize ROS production and inhibit prolonged inflammation associated with diabetic wounds [61]. Findings from an in vivo study show that ethanolic extract of propolis (EEP) improves fin regeneration in hyperglycemic-induced zebrafish [62]. The expression of genes that are involved in the protein regulation of wound healing and regeneration were also increased. In this study, the EEP powder dissolved in propylene glycol was mixed into the water tank.

*Clerodendrum cyrthophyllum Turcz* (*C. Turcz*) is a plant that has been used in folk medicine for treating a variety of inflammatory medical conditions such as migraine and rheumatoid arthritis [63]. Prior study of *C. Turcz* in zebrafish has proven its anti-inflammatory activities, which could affect the cutaneous-wound-healing process. In the study by Nguyen et al. (2020), ethanol extraction of *C. Turcz* leaves showed the ability to suppress inflammation in a tail-cut induced inflammation zebrafish model via inhibition of the eicosanoid pathway and downregulation of pro-inflammatory cytokines [64]. Another study by Nguyen et al. (2021) using the zebrafish copper-induced inflammation model confirmed the anti-inflammatory and anti-oxidant activities of *C. Turcz* via downregulation of pro-inflammatory cytokines, which limits the generation of ROS [65]. In the context of wound healing, eicosanoids play key roles in the repair of epithelial barriers in the skin [66,67].

### 4.2. Nanoparticles

Silver nanoparticles (AgNPs) have been proposed as an attracting material for surgical wound care because of their strong antimicrobial and antifungal properties [68,69]. Direct application or immersion of nanoparticles into the wound have proven to enhance wound healing in zebrafish models [37]. Wounds exposed to AgNPs have proven to stimulate the release of transforming growth factor (TGF-β), matrix metalloproteinase (MMP)-13 and -9, proinflammatory cytokines (IL-1β and TNF-α), and antioxidant enzymes superoxide dismutase (SOD) and catalase. Being antioxidants, SOD and catalase are able to regulate the concentration of ROS at the injury site to protect the cells from radical attack [70]. In these, catalase is disproportionately H_2_O_2_, while SOD is an oxidoreductase that dismutates the superoxide anion, thereby acting as signaling routes to modulate wound healing [71]. However, there is increasing concern about the possible hazardous threat of AgNPs on human health, both at the local wound and systemic organs. A study by Pang et al. (2020) suggests that AgNPs at 2 μg/mL impair the function of granulation tissue in caudal fin regeneration of the adult zebrafish model [72]. The study also suggests that the treatment with AgNPs impairs fin regeneration during the epithelialization stage and at the beginning of blastema formation. Systemically, AgNPs may be absorbed through the skin into circulation and may reach the organs such the kidney, liver, brain, and heart [73].

Spirulina is a bioactive microalga that has been incorporated into formulated and topical skincare formulas. To date, several articles have reported the promising wound healing and antioxidant properties of spirulina. In the study by Rajapaksha et al. (2020), *Spirulina maxima* derives pectin nanoparticles that are able to reduce ROS dramatically and increase immune modulatory activity in zebrafish wounds. In this case, the researchers observed that the wound-healing markers, specifically, TGF-β1, TIMP2b, MMP-9, TNF-α, IL-1, and chemokines including ccl34a.4 and ccl34b.4, were upregulated [43,74]. Resultantly, rapid wound healing, formation of the neoepidermis, and restored pigmentation were witnessed.

### 4.3. Formulated Drugs

Several formulated drugs are being tested in zebrafish to study the long-term effects in wound healing and vice versa. For instance, a detrimental effect was observed upon introducing glucocorticoid medication known as dexamethasone to a fin-amputated zebrafish model. Here, at the injury side, dexamethasone further triggers cell apoptosis and reduces immune modulatory activity and macrophages, indicating delayed wound healing [75]. Likewise, sodium warfarin and hydrocortisone application to cutaneous wounds in zebrafish impair granulation tissue formation and delay the inflammatory phase and the re-epithelialization process [76,77]. This shows that the above-tested drugs are not suitable to be applied directly to cutaneous wounds. Meanwhile, zebrafish embryos exposed with nocodazole showed increased levels of circulating neutrophils at the wounded area [78]. Nocodazole in an antineoplastic drug that interferes with the polymerization of microtubules. Microtubules, a major component of the cytoskeleton, are involved in mitosis, cell motility, and the maintenance of cell shape. In wound healing, targeting microtubules early might impact wound repair at the inflammatory and proliferative stages [76]. However, activated neutrophils protect the wound from pathogenic microbe invasion by producing proteases, ROS, and antimicrobial peptides. These substances have the ability to degrade pathogenic microbes at the wounded region, thereby accelerating the normal healing phase [77].

## 5. Advantages of Using Zebrafish as a Cutaneous Model in Wound Healing

There are numerous advantages for zebrafish being modelled for skin pathology, particularly cutaneous wound healing. This species is gaining popularity due to its cost-effectiveness and because it is an expedient species, which provides rapid results. This section will explain the advantages of using a zebrafish model in wound healing.

### 5.1. External, Transparent, and Rapid Development

Zebrafish embryos and their chorion, a protective membrane, are translucent (amniote system) and develop from an egg that has been fertilized externally. As a result, it only takes 2–3 days for a newly fertilized embryo to develop from a single cell to a free-swimming larvae. During this developmental phase, embryos at cleavage, gastrulation, and organogenesis are observable [79]. All of this phase is rapid and visually accessible. This eases the follow-up of morphological changes from day one itself. During this stage of development the epidermal wound-healing mechanism can be studied easily. When observed for 24 weeks, the mechanism of skin transition can be followed up. Interestingly, the findings are similar to the mammals’ wound-healing phase [80].

### 5.2. Large Number of Offspring and Ease of Breeding

A sexually mature, healthy female fish can produce hundreds of eggs every day, with individual clutch sizes exceeding 700 eggs. Because of its enormous reproductive capacity, the zebrafish embryo and larva are ideal for research that requires a high rate of automation. For breeding, just by placing 1–2 L of polycarbonate and the opposite gender species, the mating process can be initiated within less than 24 h. Mixture of these genders can be separated using a divider later on [81].

### 5.3. Short Reproductive Cycle

Despite having the shortest life cycle, it can regenerate a large number of tissues and organs in a short period of time. The entire life cycle, from fertilization to the production of a sexually matured organism, can take as little as 3 months [82]. In fact, this correlates with the normal healing duration. For instance, acute wound healing can heal within 4 to 6 weeks, while chronic wounds can last up to 3 months or even more. Meanwhile, the remodeling phase in mammals can take up to 12 months [25]. Additionally, they can live up to 3 years. This similarity allows researchers to study the underlying signaling mechanism and chronicity in wound healing without hesitation.

### 5.4. The Ease of Maintenance of a Large Number of Species

Zebrafish do not die easily, and they possess a gentle temperament. They even tolerate harsh environments with parameters that may differ from the ideal. An aquarium with a capacity of 10 gallons is enough to hold up to a dozen zebrafish with the temperature ranging between 18–25 °C. Since they are omnivores, feeding on small organisms within slow-moving waterways is sufficient to favor their living [83].

### 5.5. Genetics of Zebrafish

Most human genes can be found in zebrafish, and these genes have similar expression patterns. Nonetheless, their amino acid residue sequences are said to be 70% identical [84]. Surprisingly, a widespread gene duplication event appears to have happened in the zebrafish lineage, as certain zebrafish genes have duplicate orthologues. The functions of a single human gene are frequently found to be shared by the two zebrafish orthologues. In this case, if one gene is mutated or loses function, scientists can still make use of another to study the skin pathology. Moreover, these genes can be deleted using anti-sense morpholino oligonucleotides according to the study’s needs while preserving the normal function of other genes [85]. This allows researchers to investigate the role of genes involved in wound healing such as S100A, progranulin, MMP-9, αv integrin, β5 integrin, metallothioneins, and connective tissue growth factor, easily [86], particularly for drug repositories.

### 5.6. Manipulation of Genome Activity

Utilization of chemical mutagens, particularly N-ethyl-N-nitrosourea in zebrafish, allow the manipulation of gene activities and make the screening of heterogeneous genes possible [87]. Through targeted induced local lesions in a genome, isolated mutations can be grown to homozygosity. Thus, inducing the desired cleavage of DNA by introducing selected proteins is made possible [88]. This specification enables researchers to investigate how the manipulation of genes may accelerate healing, specifically for chronic non-healing wounds.

### 5.7. Ease of Drug Introduction

Drugs can be easily introduced to large-scale zebrafish at once just by placing or exposing drugs to their enclosure. Whereas, for embryos, desired drug interference can be observed by placing medication in the embryo-support medium. At the same time, distinct drugs and dosages can be tested at once using microtiter dishes [89]. This characteristic enables the use of high-throughput pharmacological screening in whole animals.

### 5.8. Test Samples for Molecular Biological Analysis

In zebrafish, injecting morpholino oligonucleotides will suppress mRNA translation. This enables researchers to identify gene misalignment during embryo development, particularly in genetic conditions [85]. This allows any errors in the molecular pathway to be detected earlier; thus, certain preventable genetic conditions might be treated earlier. Apart from this, endogenous gene expression can be studied using the same procedure. The injection can also provide a phenotypical readout of the activities of genes in disease [90]. This criterion enables us to study errors in genes related to wound healing, specifically non-healing chronic wounds.

## 6. Challenges in Handling Zebrafish as a Wound-Healing Model

Although zebrafish are an ideal model for wound healing, there are still some drawbacks. For instance, in the zebrafish developmental phase, there is no placental stage in mammals, which are termed as poikilothermic. It can be speculated that certain medications to accelerate wound healing might be metabolized via different mechanisms [91]. This means humans possess a homeothermic metabolism ability, and not all data perceived from zebrafish in vivo assays can be utilized for humans, unless done with proven clinical trials. Additionally, there are several distinct characteristics between mammals and zebrafish on the basis of gender. As a result, there is a restriction on studies relating to hormones in zebrafish [92]. In terms of the wound-healing mechanism, this statement is a major drawback. This is because a recent study claims that sex hormones play a role in the inflammatory phase, which may influence dermal wound healing [93].

Besides, imaging embryo development may require high-quality imaging due to its tiny size and shape [94]. This imaging is necessary to follow up on the cellular changes during the wound-healing process. Another possible challenge relies on then data validation between zebrafish assays and mammalian species, specifically for drug screening. Such information is required for the translation of zebrafish testing results into drug formulation that will be available for human usage. Uncertainty concerning the zebrafish model’s predictability is a primary source of skepticism [95].

## 7. Conclusions and Future Prospective

The natural characteristics of zebrafish, such as external fertilization, a large number of offspring, rapid growth, a transparent body, a common genetic phenotype, ease of experimental conduct, and an unchallenging genetic modification process, make it an excellent model to study the mechanism of cutaneous wound healing. New genomic technologies have improved the resolution of zebrafish in wound-healing mechanisms. However, they must be applied and interpreted with care. Strategic areas, such as systematic and scalable techniques of functional gene interrogation, leveraging the plethora of current models, should become a priority in order to fully realize the potential of zebrafish in wound-care research, particularly when translating to drug invention. In consideration with the scientific implications and the proven in vivo outcomes, the zebrafish model appears to have a promising future for novel discoveries.

## Figures and Tables

**Table 1:** In vivo evidence.

Author	Aim	Strain	Type of Wound	Age	Follow Up Duration	Observation	Signaling Pathway Involved
Richardson et al. (2013) [34]	To demonstrate adult zebrafish as a model for cutaneous wounds	Tg(krt4:egfp)gz7, Tg(mpx:GFP)i114, Tg(lyz:;EGFP) nz117, Tg(lyz:dsRED2)nz50, Tg(fli1a:EGFP)y1, Tg(kdrl:HSRAS:mCherry) s896, and Tg(hsp70l:dnfgfr1-EGFP)pd1	Full thickness	6–12 months	4 h–24 days	- Thin-layer neoepidermis covered the majority of the wounded region at the 6th hour post-wounding. - Multiple cell layers of the neoepidermis were formed within 24 h post-wounding. - The number of inflammatory cells started to reduce over days. - Macrophages were seen at the wounded region on the 4th day post-wounding. - Granulation tissue formation was seen as early as 24 h post-wounding. - Rapid neovascularization was seen from the 4th day post-wounding. - Decreased levels of leukocytes and blood vessels were seen from the 10th day post-wounding. - Fully re-stratified wound epidermis, dermal compartments, completely recovered subcutaneous adipocytes, scales, and pigmentation were seen on the 28th day post-wounding.	Transgenic inhibition of FGF signaling.
Lisse et al. (2016) [36]	To access the effectiveness of H_2_O_2_ in epidermal wound healing	Nacre	Epidermal	Not specified	0.5 h–4 days	- Activation of downstream pathway of reactive oxygen species (ROS). - Upregulation of MMP9 and MMP13a. - Downregulation of npas4a and serpinh1b. - Increased expression of cryptochrome (cry1). - Rapid activation of cell migration, adhesion, and cytoprotective and anti-apoptotic events. - Prolonged NF-κB activity in larvae. - Increased levels of Col5a3b and Itgb4.	Activation of EGF, FOXO1, and IKKα pathways.
Caraguel et al. (2016) [38]	To develop a differential approach in wound-healing modelling	Danio rerio	Full thickness	Not specified	2 h–14 days	- Rapid rate of re-epithelialization process. - Rapid migration of epidermal strata cell into the wound. - Re-organization of the epidermis in the basal stratum was seen within 24 h. - Cell migration in dermal was seen at 72 h post-wounding. - Increased level of EGF production in the basal epidermal. - Complete wound closure was seen by day 14.	Activation of EGF pathway.
Richardson et al. (2016) [48]	To study the underlying mechanism of cutaneous wound closure	TL, edarz^3R367W^, Tg(actb2:hras-egfp)vu119, Tg(krt4:egfp)gz7, Tg(hsp70l:EGFP), and Tg(hsp70l:dnfgfr1-EGFP)pd1	Partial and full thickness	6–12 months	30 min to 4 days	- Rapid wound closure with a speed of 500 µm/h. - Formation of bilayer, GFP+ superficial cells, and p63+ inner keratinocytes were seen. - Wound closure via the purse-string mechanism was seen. - Production of concentric rings of actin fibers and non-muscle myosin at the wound edges is seen. - Increased migration of keratinocytes to the wound region. - Increased loss of surface microridges. - Elongation of inner keratinocytes in the superficial layer.	Regulation of TGFβ/integrin- and Rock/JNK pathway.
Richardson et al. (2016) [33]	To study the role of Rho kinase (Rock) in cutaneous wound healing	Adult transgenic zebrafish	Partial and full thickness	6–12 months	1 h to 15 days	- Attachment of the inner basal layer with the basement membrane. - Outer layer was flattened and consisted of periderm cells. - Extension of lamellipodia within a few minutes. - Inhibition of TGFβ signaling enhanced keratinocytes proliferation. - Activation of receptor tyrosine kinases (RTKs) and G-protein-coupled receptors for rapid wound closure.	Activation of Rock pathway for the rapid re-epithelialization process.
Seo et al. (2017) [37]	To study the efficacy of silver nanoparticles (AgNP) for wound healing	Wild-type Danio rerio	Epidermal	4 months	2–20 days	- Absence of toxicity was seen with AgNP concentration >140 mg/L. - Faster wound closure with clear margin was seen within 5 h. - Complete filling of cavity, re-surfaced epidermal cells, well-formed skin, and immune cells is seen near the wound. - Upregulation of TGF-β mRNA in muscle. - Increased level of IL-1β, TNF-α, SOD, and catalase. - Decreased level of MMP-9 and MMP-13.	Not specified.
Xiong et al. (2018) [39]	To study the effectiveness of a collagen mixture in wound healing	Wild-type AB strain	Full thickness	Not specified	2–5 days	- Increased formation of new epithelial tissues. - Increased production of new blood vessels. - Increased level of cell proliferation at the wounded region. - Decreased level of cell apoptosis and rapid clearance of death cells at the wounded region. - Decreased levels of neutrophils after 18 h of treatment. - Suppression of the inflammatory response. - Increased expression of Msxb, vegf-A, Wnt3a, RAR, and upregulation of col1a1b.	Inhibition of TNF-mediated leukocyte chemotaxis.
Vimalraj et al. (2018) [40]	To analyze the role of nitric oxide in wound healing	Adult Tie2-GFP transgenic Zebrafish	Full thickness	8–10 months	48 h–14 days	- Rapid regeneration of fin and re-growth of blood vessel was seen. - Formation of re-epithelial cell without the lag phase was seen. - Increased level of granulated cells during early treatment. - Mitotic activity of epidermal cells was seen within 24 to 72 h. - Increased level of fibroblast synthesis was seen within the deeper layer of tissues. - Increased level of collagen deposition was seen in the wounded region.	Upregulation of the Wnt/β-catenin pathway.
Noishiki et al. (2019) [41]	To demonstrate the angiogenesis mechanism during cutaneous wound healing	Tg(kdrl:eGFP)^s843^, Tg(gata1:DsRed)^sd2^, and Tg(fli1a:mCherry)^ncv501^	Partial thickness	Not specified	2 days–2 months	- Rapid formation of endothelial cells and pericytes was seen, which lasted up to 2 months. - Activation of the angiogenesis process by endothelial cells was seen. - Increased production of new blood vessels and the bifurcation process. - Activation of VEGF signaling for angiogenesis at the wounded region was seen. - Rapid increase in pericytes covering tortuous blood vessels.	Activation of VEGF signaling pathway.
Liu et al. (2020) [42]	To investigate the role of isoliquiritin in angiogenesis during wound healing	Tg(fli-1:EGFP), and Tg(mpeg:mCherry)	Full thickness	6 months	Day 1–the 15th day	- Rapid wound closure was seen as early as the 5th day. - A decrease in wound size was seen from day 3. - Formation of a thick epithelial cell layer and degeneration of granulation tissue were seen. - Longer duration of angiogenesis was seen in the treatment group. - Increased concentration of macrophages was seen at the wounded region. - Increased expression of SOD1, TGFβ, and TNF-α were observed.	Downregulation of VEGFR tyrosine kinase inhibitor II pathway.
Edirisinghe et al. (2020) [43]	To scrutinize the ability of *Spirulina maxima* in wound healing	Wild-type AB	Full thickness	4 months	Day 1–day 10	- Increased migration of fibroblasts to the wounded region. - Rapid regeneration of fin was seen. - Rapid wound closure, re-appearance of pigments, and disappearance of wound margins at the wounded site. - Rapid process of re-epithelialization, multiple layers of neo-epidermis, and re-appearance of overlapping scales was seen. - Rapid remodeling of epidermal tissue and tissue remodeling were observed.	Upregulation of the Wnt/β-catenin pathway.

**Table 2:** Compounds with direct wound-healing effects in zebrafish models.

Agent	Model	Treatment Mode	Findings	Reference
**Natural products**				
*Panax ginseng*	Tail fin amputation in zebrafish larva	Incubation medium	Potent anti-inflammatory effects similar to beclomethasone No effect on regeneration	[53]
*Panax ginseng*	Zebrafish embryos	Incubation medium	Promotes angiogenesis Increase in sub-intestinal vascular growth (dosage 500 μg/mL)	[56]
*Curcuma longa*	Caudal fin transection in adult zebrafish	Topical application	Maximum fin regeneration with 500 μg of the aqueous extract on day 5 Drastic neutrophil migration reduction on day 5	[57]
Ethanol extract Propolis (*Trigona laeviceps*)(EEP)	Caudal fin amputation in hyperglycemia model (induced by alloxan and glucose) in adult zebrafish	Water immersion	Increased fin regeneration Increased gene expression involved in wound healing and regeneration (shha, igf2a, bmp2b, and col1a2)	[62]
**Nanoparticles**				
Silver nanoparticles	Caudal fin regeneration model in adult 3-month-old zebrafish	Water immersion	Increased fin regeneration Increased gene expression involved in wound healing and regeneration	[72]
Silver nanoparticles	Laser-induced wound injury, posterior to the gill area in adult zebrafish	Water immersion and direct skin application	Faster wound closure at 5, 10, and 20 dpw by both AgNPs application methods AgNPs by immersion have a higher wound-healing activity Expression of wound-healing genes (TGF-β and MMP -13 and -9), proinflammatory cytokines (IL-1β and TNF-α), and antioxidant enzymes SOD and catalase	[37]
*Spirulina maxima*-derived pectin nanoparticles (SmPNPs)	Laser-induced wound at the left flank in adult zebrafish	Direct skin application	Rapid wound closure at 7, 10, 14, and 24 dpw Significant wound healing percentage compared to the vehicle at 10 dpw Histologically, rapid re-epithelialization in the SmPNPs-treated group at 7 dpw Upregulated wound-healing marker in SmPNPs treated group (*tgfβ1, timp2b, mmp9, tnfα, il1β, ccl34a.4, and ccl34b.4*)	[74]
*Spirulina maxima*-derived marine pectin (Smp)	Fin regeneration model in zebrafish larvae; laser-induced wound in adult zebrafish	Topical application	Enhanced fin regeneration upon Smp treatment at 3 dpw in zebrafish larvae SMP accelerate open-skin wound closure in adult zebrafish SMP-treated fish exhibited rapid re-epithelization at 2 dpw Upregulation of wound-healing marker (*tgfβ1, timp2b, mmp9, tnfα, il1β, ccl34a.4, and ccl34b.4*)	[43]
**Formulated drug**				
Nocadazole	Caudal fin amputation in zebrafish embryos	Incubation medium	Nocodazole significantly increased the circulatory neutrophils at the wounded fin	[78]

## Data Availability

Data is contained within the article.
